# Efficacy of lapatinib monotherapy on occult breast cancer presenting with cutaneous metastases: A case report

**DOI:** 10.3892/ol.2014.2594

**Published:** 2014-10-09

**Authors:** EIICHIRO NOGUCHI, TAKAKO KAMIO, HIDENORI KAMIO, HIROKO MIURA, MASAKO TAMAKI, MASAKO NISHIZAWA, KEI AOYAMA, TETSUYA OOCHI, SHINGO KAMEOKA

**Affiliations:** Department of Surgery II, Tokyo Women’s Medical University, Tokyo 162-8666, Japan

**Keywords:** occult breast cancer, lapatinib monotherapy, cutaneous metastases, serum human epidermal growth factor receptor 2

## Abstract

The case of a 72-year-old female who identified a lymph node enlargement in the left axilla is reported in the present study. A lymph node biopsy revealed a metastatic adenocarcinoma of the axillary lymph node. Following various assessments, the patient was diagnosed with occult breast cancer and lymph node metastases, for which treatment was initiated. Trastuzumab monotherapy was administered as the patient was elderly, positive for the hepatitis B virus and exhibited the following immunostaining/immunohistochemical analysis results: Estrogen receptor (ER) negative (−), progesterone receptor (PgR) negative (−) and human epidermal growth factor receptor 2 (HER2) positive (3+). Breast ultrasonography was performed 10 months after the initial trastuzumab administration and the left axillary lymph node enlargement had reduced in size and severity. However, a skin rash (erythema) was observed encompassing the left breast and extending into the axilla. As determined by the result of a skin biopsy of this area, the patient was diagnosed with occult breast cancer with cutaneous metastases. The immunohistochemical analysis results obtained from the skin biopsy were similar to those obtained from the lymph nodes: ER (−), PgR (−) and HER2 (3+). Therefore, the patient was switched from trastuzumab to lapatinib monotherapy. The erythema completely disappeared after two months of treatment. At present (34 months following lapatinib monotherapy initiation) no new lesions or severe side-effects have been observed.

## Introduction

Trastuzumab, an anti-human epidermal growth factor receptor 2 (HER2) monoclonal antibody and lapatinib, an epidermal growth factor receptor (EGFR) and HER2 tyrosine kinase inhibitor were the only two anti-HER2 agents that had been approved in Japan as of September 2013. Administration of anti-HER2 agents in combination with chemotherapy is the first-line treatment for patients presenting with HER2-type breast cancer (1–4). However, in the case outlined in the present study, the patient was elderly and had a history of hepatitis B (HB); therefore, discarding the use of chemotherapy was considered and the patient was treated with trastuzumab monotherapy. However, after the patient was assessed as achieving clinical partial response, cutaneous metastases developed, which resulted in disease progression. However, clinical complete response (cCR) was sustained in the long-term subsequent to switching to treatment via lapatinib monotherapy. In the present study, the clinical course of a breast cancer patient is reported. Written informed consent was obtained from the patient.

## Case report

In December 2009, a 72-year-old female was admitted to the Tokyo Women’s Medical University (Tokyo, Japan) presenting with a mass in the left axillary lymph node. The patient had been diagnosed with HB (cause unknown) at the age of 37 years. During medical treatment at the age of 52 years, the following antigen loss was observed: Hepatitis B surface (HBs) antigen negative (−), HB virus (HBV) DNA real-time (−), HBs antibody positive (+) and Hepatitis B core antibody (+). The patient’s older sister had been diagnosed with breast cancer, a younger sister suffered from pancreatic cancer and a younger brother had esophageal cancer. In November 2009, the patient presented with a mass in the left axilla and visited Fukujuji Hospital (Tokyo, Japan). A mass biopsy was performed and the patient was diagnosed with metastatic adenocarcinoma of the axillary lymph node.

**Figure 1:**
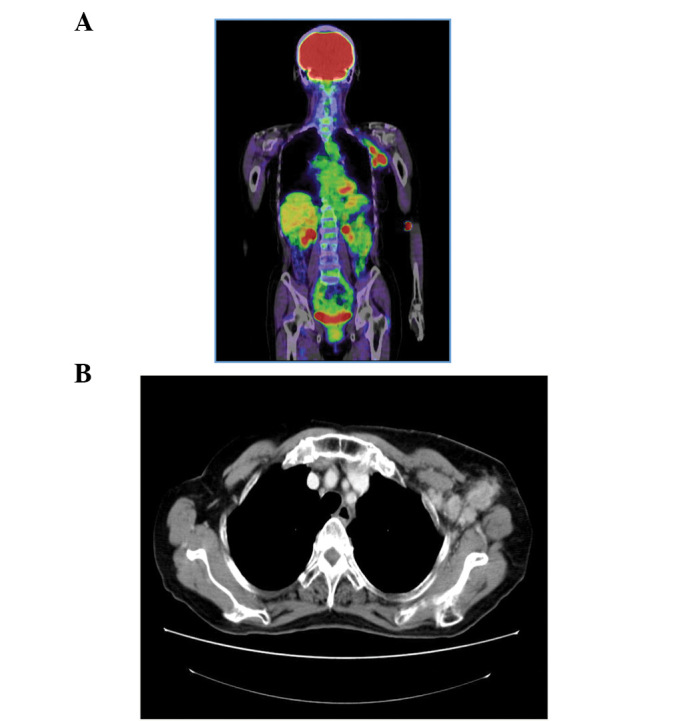
Position electron tomography (PET) and contrast-enhanced computed tomography (CT). (A) Fludeoxyglucose (FDG)-PET: FDG accumulation only in the left axillary lymph node. (B) Contrast-enhanced CT: Swelling in the left axillary lymph node.

The primary tumor was not detected during fludeoxyglucose-position electron tomography (Fig. 1A). In addition, no primary tumor was observed by chest computed tomography (CT; Fig. 1B), abdominal-pelvic CT or via a gynecological screening. Therefore, the patient was referred to Tokyo Women’s Medical University hospital to undergo a breast cancer screening.

**Figure 2:**
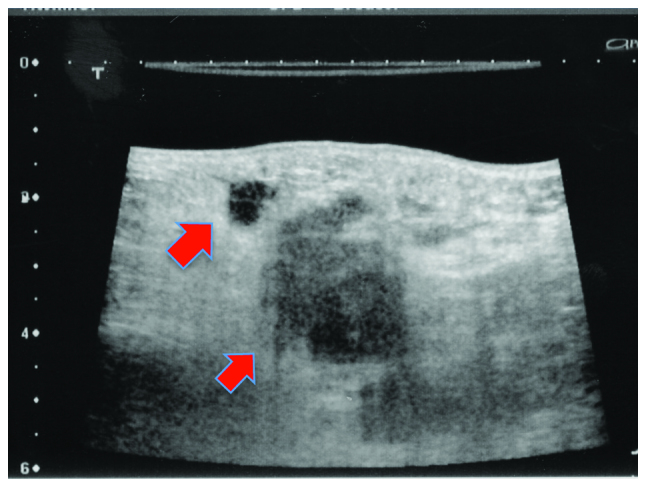
Breast ultrasonography of the axillary lesion. A minimum of 20 irregular masses of dispersed sizes were observed surrounding the suspected metastatic left axillary lymph node. The smallest and greatest diameters observed were 5 and 35 mm, respectively.

Upon clinical breast examination, no evidence of skin retraction or nipple dimpling was observed. In addition, there was no apparent evidence of a mass in the mammary gland. However, the breast cancer screening revealed a well-defined 30-mm diameter non-movable mass in the left axillary lymph node. A mammography did not reveal any abnormalities on either side and during breast ultrasonography, no marked tumor lesions were detected in the mammary gland. A suspected metastases (maximum diameter, 35 mm) was observed in a lymph node in the left axilla (Fig. 2) and a minimum of 20 irregular masses of varying sizes were observed in this area. No evidence of metastases was detected in the lymph nodes proximal to the sternum and clavicle fossae.

When analyzing the tumor markers (Table I) a marginal increase from the standard value in serum HER2 levels was detected, however there were no increases observed in the other markers.

**Figure 3:**
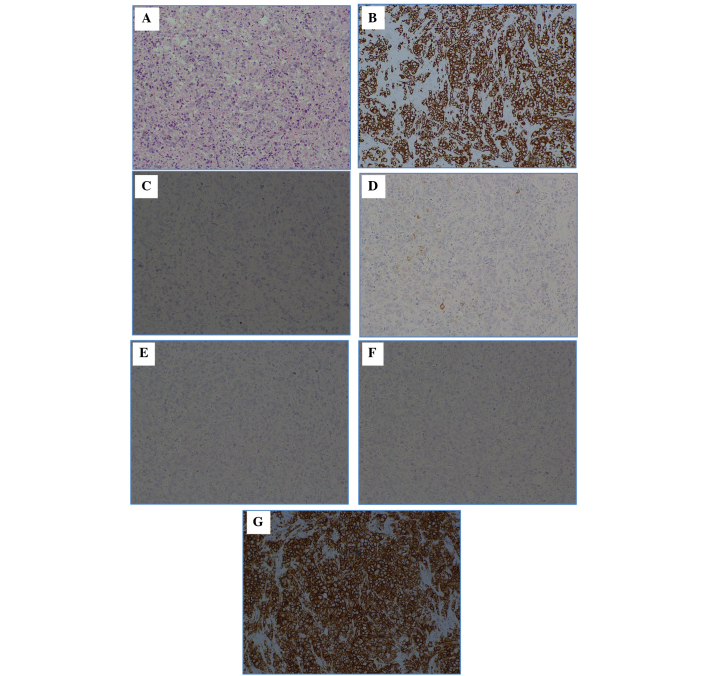
Hematoxylin and eosin (H&E) staining, and various types of immunostaining of the left axillary lymph node mass. (A) H&E; (B) cytokeratin (CK)7; (C) CK20; (D) gross cystic disease fluid protein 15 (GCDFP15); (E) estrogen receptor (ER); (F) progesterone receptor (PgR); and (G) human epidermal growth factor receptor 2 (HER2). The following immunostaining results were obtained: CK7 positive (+), CK20 negative (−), GCDFP15 (partial +), ER (−), PgR (−) and HER2 score (3+). Magnification, ×100.

Pathological analyses were conducted on the left axillary lymph node mass (Fig. 3). In the lymph node, hematoxylin and eosin staining detected a metastatic carcinoma and the immunostaining results were as follows: Cytokeratin (CK)7 (+), CK20 (−), gross cystic disease fluid protein 15 (partial +), estrogen receptor (ER; −) and progesterone receptor (PgR; −). The lungs, mammary glands, ovary, uterus, and mesothelium was hypothesized to have primary lesions and the HER2 score was 3+.

However, no primary lesions were identified, including in the mammary gland. The most frequent type of malignant tumor exhibiting only axillary lymph node enlargement is breast cancer, which accounts for >50% cases, worldwide (5–8). The primary occult breast cancer lesion is not identified in 0.3–1.0% of resectable breast cancer cases, worldwide (5). The patient in the current case study was treated for occult breast cancer as a result of the immunohistochemical staining results and the presentation of axillary lymph node metastases alone (T_0_N_2_M_0_; Stage IIIA) (9). Primary systemic therapy was considered, as the patient was Stage IIIA. However, as the patient had a history of HBV infection, there was a risk of *de novo* HB development due to administration of anticancer agents. This, in addition to the age of the patient, resulted in the patient being initially treated with trastuzumab monotherapy.

**Figure 4:**
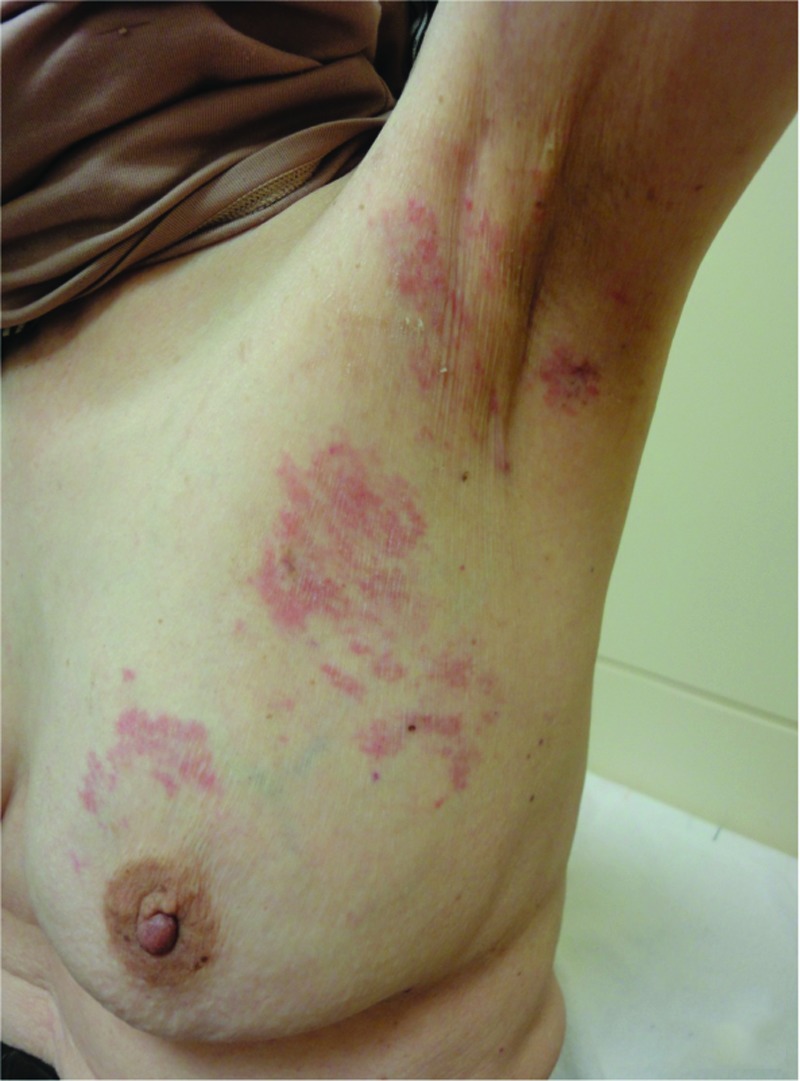
Cutaneous metastases. December 2010: Flat polypoid lesions of eczema appeared in the area between the upper left breast and axilla.

**Figure 5:**
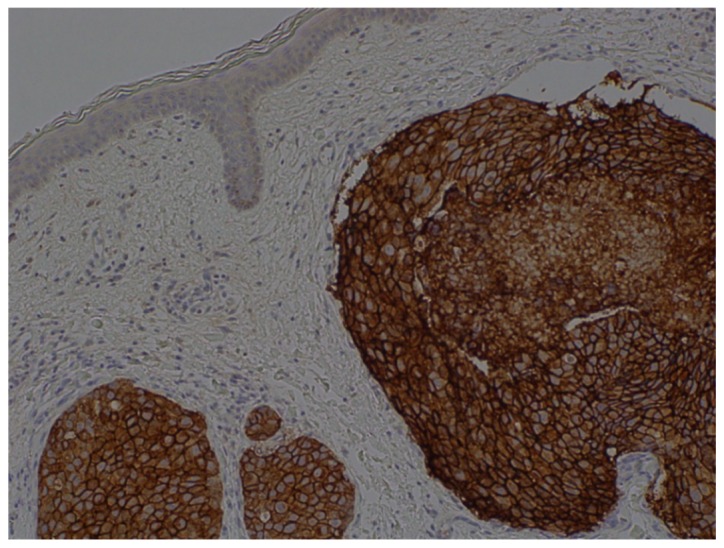
Histopathological observations of a skin biopsy (human epidermal growth factor receptor 2 staining; 3+) revealed an adenocarcinoma. Atypical cells with large nuclei were detected in the blood and lymphatic vessels of the dermis and the vessels contained atypical cells (magnification, ×100).

**Figure 6:**
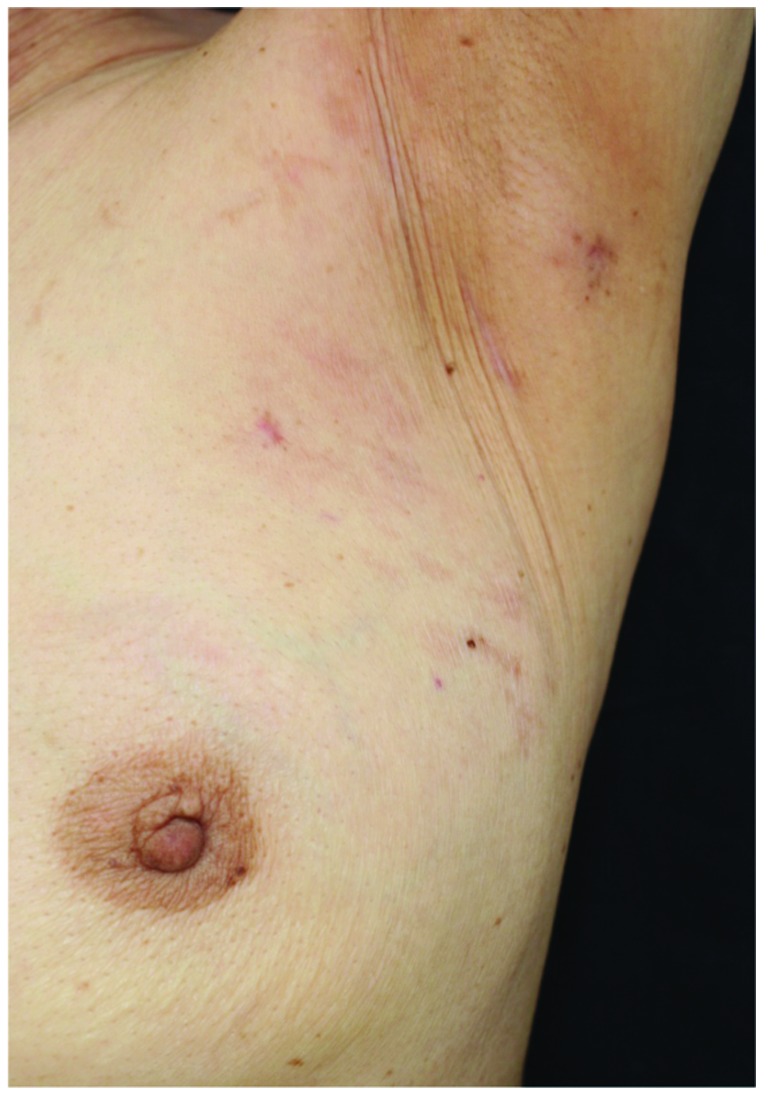
Cutaneous metastases. March 2011: The rash was barely noticeable 21 days after lapatinib treatment initiation. April 2011: The rash disappeared.

Trastuzumab was administered as a monotherapy from February 2010. Mammary ultrasonography was performed 10 months following the initial trastuzumab administration, and the irregular masses in the left axillary lymph node reduced in size and number. However, a skin rash (erythema) was observed encompassing the left breast and extending into the axilla (Fig. 4). The pathological findings from the skin biopsy of the affected area, as well as the immunostaining findings, were comparible with those of the malignant lymph nodes (Fig. 5). Therefore, the patient was diagnosed with occult breast cancer with cutaneous metastases. In February 2011, administration of the anti-HER2 agent was switched from trastuzumab to lapatinib. The erythema completely disappeared following two months of lapatinib administration (Fig. 6).

**Figure 7:**
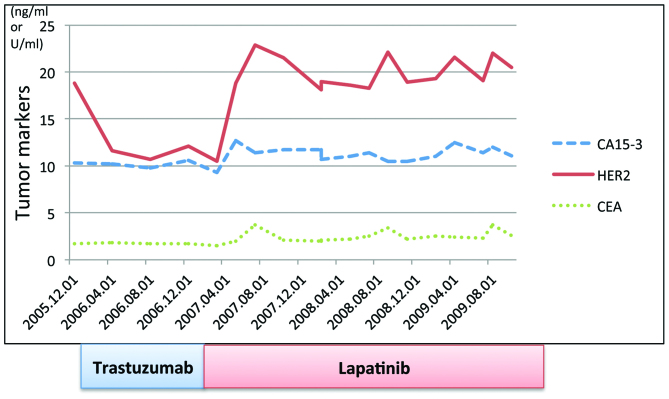
Treatment progress. Transition and progression of tumor marker levels. February 12, 2010: Initiation of trastuzumab administration. The HER2 serum concentration immediately declined to below the reference value following initiation of therapy. December 2010: A patch of eczema appeared in the area between the upper left breast and axilla, which was identified as skin metastases; the HER2 serum concentration was normal when this occurred. February 19, 2011: Initiated lapatinib administration. Skin metastases disappeared two months after switching to lapatinib; however, the HER2 serum concentration was 18.8 ng/ml, which exceeded the reference value (15.2 ng/ml). Thereafter, no evident new lesions were observed, and the HER2 serum concentration was maintained between 18.1 and 22.9 ng/ml, marginally greater than the reference value. CA15-3, cancer antigen 15-3; HER2, human epidermal growth factor receptor 2, CEA, carcinoembryonic antigen.

At present, 34 months following initiation of lapatinib treatment, no new lesions or severe side-effects have been observed. Fig. 7 illustrates the progress during the treatment period.

## Discussion

Accounting for the extent of the axillary lymph node metastases, the standard treatment for the patient in the present case study would usually have been neoadjuvant chemotherapy via administration of anticancer agents, such as anthracycline and taxane (plus the anti-HER2 agent, trastuzumab), followed by surgery and radiation therapy, followed up with administration of trastuzumab as an adjuvant therapeutic agent. However, the decision to initiate treatment using trastuzumab as a monotherapy was determined based on the following factors: i) The patient had a history of HBV infection and there was a risk of developing *de novo* HB as a result of administering the anticancer agents (10) and ii) the patient was elderly (age, 72 years). Although cCR in the axillary lymph node had almost been reached 10 months subsequent to the initiation of trastuzumab administration, cutaneous metastases appeared in the skin in the area between the upper left breast and axilla. Distant metastases were not observed in any of the other areas. However, the extent of skin metastases had spread to the medial side of the arm beyond the axillary side. The severity reached a level such that skin grafting was required via radical surgery. Therefore, the anti-HER2 agent, trastuzumab was replaced with lapatinib monotherapy. The skin metastases disappeared after two months of lapatinib administration and the patient was considered to be in cCR. Currently, 34 months subsequent to lapatinib administration, no new lesions or severe side-effects have been observed.

When the tumor markers were examined in the present case study, the serum HER2 levels exhibited the only variation. HER2 serum concentration is a measurement of the serum extracellular domain (ECD) level. HER2/neu proteins located in the ECD are cleaved and separated by shedding, and remain as ECD in the serum. The upper limit of normal serum HER2 levels is defined as 15.2 ng/ml (11).

In the present case study, the HER2 serum concentration level at the time of the initial visit was 18.8 ng/ml. This level was immediately reduced to the reference value subsequent to trastuzumab treatment initiation. However, the HER2 serum concentration was at the normal value when the skin metastases presented. Two months after switching to treatment with lapatinib, when skin metastases had disappeared, the HER2 serum concentration was 18.8 ng/ml, which exceeded the reference value (15.2 ng/ml). However, since then, no apparent new lesions have been observed, and the HER2 serum concentration has been maintained between 18.1 and 22.9 ng/ml, which is marginally greater than the reference value. Scaltriti *et al* (12) reported that the HER2 protein expression levels were reduced following trastuzumab administration in mice; by contrast, the HER2 protein expression levels were increased subsequent to lapatinib administration.

In the present case, there were no observations that indicated cancer recurrence. This may have been due to the lapatinib administration, whereby occult breast cancer cells, including circulating tumor cells and other cells observed in normal tissue, such as in the myocardial cell, induced the HER2 proteins.

Trastuzumab and lapatinib were the only two anti-HER2 agents that had been approved in Japan as of September 2013. Anti-HER2 agents may be administered in the following treatment strategies: i) Anti-HER2 agent plus chemotherapy; ii) anti-HER2 agent administered as a monotherapy (13–15); and iii) anti-HER2 agent plus hormonal therapy (16,17). The current recommendation for the first-line therapy is an anti-HER2 agent plus chemotherapy. However, in the present case study, the administration of an anti-HER2 agent as a monotherapy was controversial due to the patient’s HER2 subtype, history of HB and other such characteristics.

**Figure 8:**
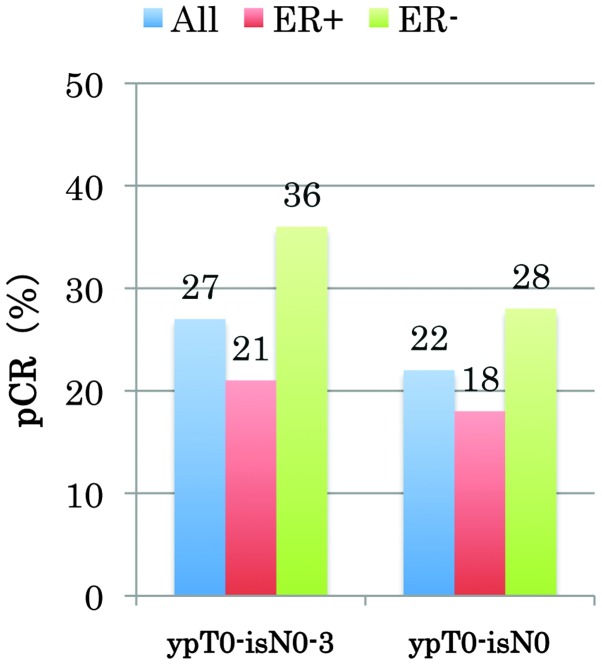
TBCRC 006 phase II trial. ER, estrogen receptor; pCR, complete pathological response. ypT0-isN0, no residual invasive cancer in the breast or axilla; ypT0-N0-3, no residual invasive cancer in the breast.

The TBCRC 006 trial (18), a single-arm phase II study, is a clinical trial of neoadjuvant lapatinib and trastuzumab with hormonal therapy and without chemotherapy in patients with HER2-overexpressing breast cancer using only the two anti-HER2 agents [combined with hormonal therapy, in the case of the ER (+) subjects]. The complete pathological response rate for ER (−) (HER2-type) was high at 28% (Fig. 8) (18). In the present case, the treatment was switched to lapatinib when the patient demonstrated resistance to trastuzumab. Therefore, the results of the abovementioned clinical trial cannot be directly applied. However, the present results demonstrate that a type of breast cancer exists upon which anti-HER2 monotherapy exerts an effect. Pertuzumab, which is a HER dimerization inhibitor, has become available in Japan (19,20). Identifying biomarkers of this type of breast cancer, which exhibits a CR as a result of the administration of anti-HER2 agents only, may significantly alter the treatment strategies that are adopted for HER2-type breast cancer.

In conclusion, in the present study, a case of a patient presenting with occult breast cancer and cutaneous metastases, who was successfully treated with lapatinib monotherapy and who sustained cCR in the long-term is reported. After a total of 34 months following the initiation of lapatinib monotherapy, no novel lesions or severe side-effects have been identified.

The results of the current study may lead to the development of numerous anti-HER2 agents. Future studies are required to identify biomarkers of HER2-type breast cancer, which may be used as monotherapy agents.

## Figures and Tables

**Table I:** Tumor markers examined during the patient’s initial visit.

Tumor marker	Serum level	Standard value
CEA (ng/ml)	1.7	≤5.0
CA15-3 (U/ml)	10.3	<25
BCA225 (U/ml)	47.0	<160
NCC-ST-439 (U/ml)	4.2	<7.0
Serum HER2 protein (ng/ml)	18.8	≤15.2
Serum p53 antibodies (U/ml)	≤0.4	≤1.3

CEA, carcinoembryonic antigen; CA15-3, cancer antigen 15-3; BCA225, breast cancer antigen 225; HER2, human epidermal growth factor recpetor 2.
